# Design of the formalized and integrated Alzheimer’s Disease Ontology and its application in retrieving textual data via text mining

**DOI:** 10.1093/database/baad085

**Published:** 2023-12-02

**Authors:** Bide Zhang, Vanessa Lage-Rupprecht, Philipp Wegner, Astghik Sargsyan, Stephan Gebel, Marc Jacobs, Jürgen Klein, Martin Hofmann-Apitius, Alpha Tom Kodamullil

**Affiliations:** Department of Bioinformatics, Fraunhofer Institute for Algorithms and Scientific Computing (SCAI), Schloss Birlinghoven, Sankt Augustin 53754, Germany; Department of Bioinformatics, Fraunhofer Institute for Algorithms and Scientific Computing (SCAI), Schloss Birlinghoven, Sankt Augustin 53754, Germany; Department of Bioinformatics, Fraunhofer Institute for Algorithms and Scientific Computing (SCAI), Schloss Birlinghoven, Sankt Augustin 53754, Germany; Department of Bioinformatics, Fraunhofer Institute for Algorithms and Scientific Computing (SCAI), Schloss Birlinghoven, Sankt Augustin 53754, Germany; Department of Bioinformatics, Fraunhofer Institute for Algorithms and Scientific Computing (SCAI), Schloss Birlinghoven, Sankt Augustin 53754, Germany; Department of Bioinformatics, Fraunhofer Institute for Algorithms and Scientific Computing (SCAI), Schloss Birlinghoven, Sankt Augustin 53754, Germany; Department of Bioinformatics, Fraunhofer Institute for Algorithms and Scientific Computing (SCAI), Schloss Birlinghoven, Sankt Augustin 53754, Germany; Department of Bioinformatics, Fraunhofer Institute for Algorithms and Scientific Computing (SCAI), Schloss Birlinghoven, Sankt Augustin 53754, Germany; Department of Bioinformatics, Fraunhofer Institute for Algorithms and Scientific Computing (SCAI), Schloss Birlinghoven, Sankt Augustin 53754, Germany; Causality Biomodels, Kinfra Hi-Tech Park, Kalamassery, Cochin, Kerala 683503, India

## Abstract

As one of the leading causes for dementia in the population, it is imperative that we discern exactly why Alzheimer’s disease (AD) has a strong molecular association with beta-amyloid and tau. Although a clear understanding about etiology and pathogenesis of AD remains unsolved, scientists worldwide have dedicated significant efforts to discovering the molecular interactions linked to the pathological characteristics and potential treatments. Knowledge representations, such as domain ontologies encompassing our current understanding about AD, could greatly assist and contribute to disease research. This paper describes the construction and application of the integrated Alzheimer’s Disease Ontology (ADO), combining selected concepts from the former version of the ADO and the Alzheimer’s Disease Mapping Ontology (ADMO). In addition to the existing entities available from these knowledge models, essential knowledge about AD from public sources, such as newly discovered risk factor genes and novel treatments, was also integrated. The ADO can also be leveraged in text mining scenarios given that it is conceptually enriched with domain-specific knowledge as well as their relations. The integrated ADO consists of 39 855 total axioms. The ontology covers many aspects of the AD domain, including risk factor genes, clinical features, treatments and experimental models. The ontology complies with the Open Biological and Biomedical Ontology principles and was accepted by the foundry. In this paper, we illustrate the role of the presented ontology in extracting textual information from the SCAIView database and key measures in an ADO-based corpus.

**Database URL:**  https://academic.oup.com/database

## Introduction

### Dementia and Alzheimer’s Disease

With over 55 million patients and 10 million new incidences annually, dementia results in considerable health, social and economic costs (1). Alzheimer’s disease (AD) is the most common form of dementia affecting 60–70% of the confirmed dementia cases (1, 2). Cognition function deterioration and memory loss are characterized as the most common symptoms in AD patients regardless of the stage of progression (3). Thanks to decades of dedicated research, clear molecular dispositions associated with AD have been identified: neurofibrillary tangles and senile amyloid beta plaque accumulation in patient brains (4, 5). The key player whose abnormal aggregation leads to the formation of neurofibrillary tangles is the tau protein, while amyloid beta forms the senile plaques observed in patients (6, 7). The histological findings regarding these two misfolded proteins hallmarked the understanding of AD, although the exact mechanism of their formation and relation to pathogenesis remains elusive (8). Numerous research studies contributed to understanding the neurodegenerative disease over the past few decades, including pathological, etiological and therapeutic perspectives (9, 10). However, the present data from the field lack an integration (11) formulated in interoperable structures, supporting the overall readability and incorporating relational aspects of the existing knowledge in a common semantic interpretation. Building bridges between data integration and knowledge discovery will improve research efforts by providing standardization and operational efficiency.

### Formal ontology and biomedicine

Mohammad and Amman (12) define an ontology as ‘a formal, explicit specification of a shared conceptualization of a domain’. It can create an explicit knowledge representation through conceptualization and formalization regardless of the field of study it is describing (13). Web Ontology Language (OWL) is the semantic language designed to represent knowledge about things which computer programs can exploit (https://www.w3.org/2001/sw/wiki/OWL). Classes, properties and instances from the OWL format are terminologically modulated from concepts, roles and individuals from SROIQ description logic. Importantly, the ‘object’, ‘data’ and ‘annotation’ properties are featured in the OWL ontology (14), providing a means to feature interaction instances and hierarchical relations among terms, assign literal value to those terms (14, 15) or annotate attributable entities with labels. To a certain extent, OWL-based ontologies can resemble the knowledge that we understand in reality through its hierarchical format and various properties and thus can be used as a reliable tool for knowledge representation of the vast pool of information (16). These knowledge models represent the concepts from the perspective of one specific domain and can be applied in various ways, such as document retrieval (17), free-text processing (18–20) and text mining (21).

Diverse levels of formalization exist for formatting ontologies in different domains (22). The ontologies designed for biomedical fields maintain a certain level of formality (23). Formal ontologies have reasonableness (24), and formalisms of ontologies contribute to semantic readability and interoperability among the ontological models in the community. In an ideal scenario, the terms with the same semantic meaning derived from different biomedicine-oriented ontologies should be identical or share similarities to a great extent that interoperate between ontologies or even cross-reference in other models, which could further extend the border of knowledge representation and application in a broader range.

The ontology community favors domain ontologies with a certain formality. Moreover, many ontologies have approached a primary alignment with Basic Formal Ontology (BFO) to improve interoperability further and better represent the knowledge hierarchy (https://basic-formal-ontology.org/). BFO is a small and upper-level ontology that solely contains general concepts understood from philosophical perspectives without touching scientific terminologies, supporting dozens of applied ontologies since its initiation in 2002 (25). As one of the recommendations for submissions to the Open Biological and Biomedical Ontology (OBO) (26) foundry, this top-level ontology with systemic and fundamental structure provides a vehicle for biomedical research by which a network of multiple ontologies that might be distantly linked yet share the same ‘scaffold’ can be built (27).

### Alzheimer’s Disease Ontology and Alzheimer’s Disease Mapping Ontology

The original version of Alzheimer’s Disease ontology (ADO) developed by Ashutosh Malhotra *et al.* (28) demonstrated the first attempt at integrating AD-related information, including preclinical, clinical, pathological and associated molecular/cellular mechanisms valuable for AD-oriented research. The original ADO was created by combining disease-specific knowledge via web-based sources. Interoperability between the original ADO and other ontologies was partially accomplished through alignment with selective BFO terms.

Similarly, Alzheimer’s disease map ontology (ADMO) is a model covering knowledge specific to AD research (29). ADMO was developed based on the disease map (DM) Alzpathway (30) in AD research. The key protein interactome and their different states led by post-translational modifications in AD-related pathways are loudly described in Alzpathway. This knowledge was captured and stored in ADMO for ontological representation. In particular, ADMO has converted relations in Alzpathway into attributed ontological concepts supported by Systems Biology Ontology upper classes (29). OBO Foundry offers a platform for the ontological community and follows principles contributing to scientifically accurate and interoperable ontologies (26). The updated ADO ontology was primarily developed on top of the selective knowledge derived from the original ADO, additionally in compliance with the OBO Foundry principles, which makes it possible to align with other domain ontologies and to increase interoperability within the OBO community. To further expand the coverage of knowledge about AD in the updated ADO, we aim to consolidate and transfer the knowledge from ADMO and unify the usage of controlled vocabulary, ensuring integrity and consistency in the updated ontology.

While investigating the entities in ADMO, we discovered that the relational classes derived from ADMO possess high specificity in their form, interaction and association with the other molecules. These concepts are difficult to find and reuse due to weak interoperability. To address this, and to align with controlled vocabularies, these classes were decomposed in a way that the original form of the molecules and their interaction types were taken separately and reformulated into single classes. Reformatting these terms has allowed a stronger connection of ADMO terms with the other ontologies through small and semantically readable classes and has maximized the coverage of knowledge retrieval during text mining while maintaining the specific relations between molecules essential in AD pathways.

In this paper, we describe the construction of the updated ADO, which includes manually curated terms from ADO and ADMO and the knowledge derived from journals, web sources and books. The extensional contents from the additional sources ensure coverage of the most relevant risk factor genes, disease stages and diagnostic perspectives in AD research to date. Our work aims to integrate the information mentioned earlier and formulate the newest version of ADO compatible with the operability of the other (OBO) ontologies. In later sections, we illustrate one of the use case scenarios of ADO and its sub-ontologies, which have been used as text mining bins for tagging and filtering documents in the SCAIView (31) database.

## Results

The updated version of ADO (http://purl.obolibrary.org/obo/ado.owl, version 2.0.1) has been constructed and is ready for application. In summary, with a total count of 39 854 axioms, 1963 classes (1910 classes imported from other OBO ontologies and 53 original class axioms) and 12 object properties, the updated ADO contains relevant knowledge ranging from research, preclinical, clinical and molecular interactions and mechanisms to diagnostics, study types and treatments.

### Alignment with OBO

Selected BFO terms were included in the updated version of ADO, assuring a similar upper-class structure as the other OBO ontologies. Subsequently, we manually curated the entities found in the former version of ADO and searched for their availability in other ontologies in OBO format. Until now, we have included the data retrieved from a range of ontologies. Table 1 shows the most disease-related ontologies and respective number of classes imported to ADO. This gives a snapshot about reusing OBO classes and ensures interoperability of ADO with other ontologies.

**Table 1.** The most relative ontologies in OBO format used in updated ADO

Ontology	Class count
BFO	36
The Braunschweig Enzyme Database (BRENDA) Tissue Ontology	8
Chemical Entities of Biological Interest	347
Cell Ontology	33
Cell Line Ontology	2
Core Ontology of Clinical Trials	8
Human Disease Ontology	44
The Drug Ontology	9
Gene Ontology	138
Human Phenotype Ontology	22
Mental Disease Ontology	2
Mondo Disease Ontology	36
Protein Modification	1
Ontology for Biomedical Investigations	19
Ontology of Genes and Genomes	532
Ontology for General Medical Science	21
PRotein Ontology	7
OBO Relations Ontology (RO)	10
Symptom Ontology	32
UBERON	103

An illustration of the number of class axioms derived from OBO ontologies. ADO integrates AD-related knowledge found in 62 ontologies to ensure interoperability. This table only demonstrates the most disease-related ontologies out of all ontologies used in building ADO.

**Figure 1.**
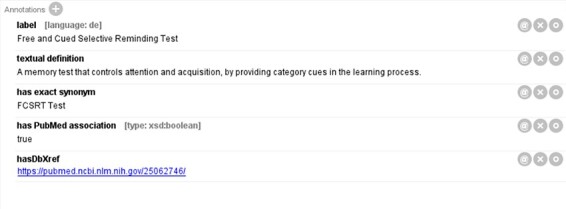
An original term ‘Free and Cued Selective Reminding Test’ defined in the integrated ADO.

Meanwhile, we have observed that a few terminologies essential to AD are not available in any of the OBO ontologies registered on the Ontology Lookup Service (OLS) (32). To address this issue, our internal team defined, annotated and curated these terminologies. Figure 1 illustrates a newly defined term, ‘Free and Cued Selective Reminding Test’, in the integrated ADO. The term was annotated with a definition, a synonym and a reference link. A similar procedure was followed for annotating the other 35 terminologies from the previous ADO ontology that were not found on OLS.

Finally, the integrated ADO was enriched with the most up-to-date and relevant knowledge of AD, derived from Alzforum (https://www.alzforum.org/), Alzheimer’s Association (https://www.alz.org/) and PubMed publications. Since most of these terms were not present on the OLS platform, we created the corresponding terms with appropriate naming (controlled vocabulary) and definition strictly following the OBO principle (e.g. definition with genus-differentia format). These terminologies were placed under the most suitable parent classes in the hierarchy. Finding the parent classes could be challenging, yet we genuinely tried to analyze how a similar term was classified in OLS and attempted to adopt the same idea for our original terminologies. In total, we created 27 original classes based on the AD-related knowledge retrieved from the sources mentioned earlier.

### Selection of terminologies and relation processing

Importing the relevant terminologies from existing knowledge models in a developing ontology complies with the reuse principle within the ontology community. Before constructing the integrated version of ADO ontology, we carefully examined the former version of ADO and ADMO. We developed specific criteria about whether the corresponding terms should be included in the integrated version of the ontology. The first step was to filter based on whether the entity is a controlled vocabulary. In the former ADO, several terms, such as ‘thing that can increase the risk, ‘Microglia cell line related to non-clinical entity’ or ‘Micro RNA from PBMC cells’, incorporate situationally specific phrases. These phrases are challenging to define and could cause a reduced recall rate in text mining due to their lower generalizability and inconsistent usage across publications. These terms were not considered part of the integrated version of ADO.

Furthermore, we excluded the obsolete classes from the original ADO to maintain clarity. The obsolete classes present in the original ADO ontology can be exemplified by ‘processual_entity’, which has now been removed by its original Uber-anatomy ontology (UBERON) ontology. Another criterion for term selection was if it would cause redundancy in our model. We attempted to avoid overcrowded classes in the intermediate levels while maintaining the necessary hierarchical classification. For example, the integrated knowledge model benefited from the removal of terms like ‘drug used in treatment’ or ‘clinical context’ in maintaining concise expression. Lastly, the relevance of these classes was investigated. Previous ADO included classes like mouse strains (e.g. ‘Lrp1tm2Her’ and ‘Klc1tm1Gsn’) for Alzheimer’s studies, migrant information (‘first-generation migrant’ and ‘second-generation migrant’) or prevalence-related measurements (‘age-specific mortality rate’ and ‘age-standardized mortality rate’), among others. These entities were considered irrelevant to the scope of the integrated ADO ontology and, therefore, were not taken as a part of the knowledge integration. In total, 107 classes with redundancy, 24 obsolete classes, 46 irrelevant classes and 87 uncontrolled vocabularies were not selected from the original ADO during the construction of the integrated version.

The relevance of ADMO classes was evaluated subsequently. ADMO concentrates the pathway and interactome knowledge. During the process of integration, we were aware that the addition of all the genetic and protein relations from ADMO would shift the focus of the integrated ontology toward molecular interactions. Thus, we only included the interactome knowledge related to the risk factor genes in the current Alzheimer‘s studies.

**Figure 2.**
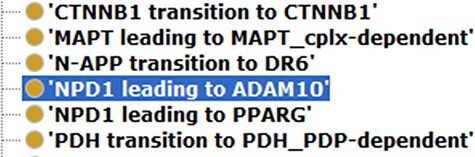
An example of ADMO terms that describe the relations between molecules.

**Figure 3.**
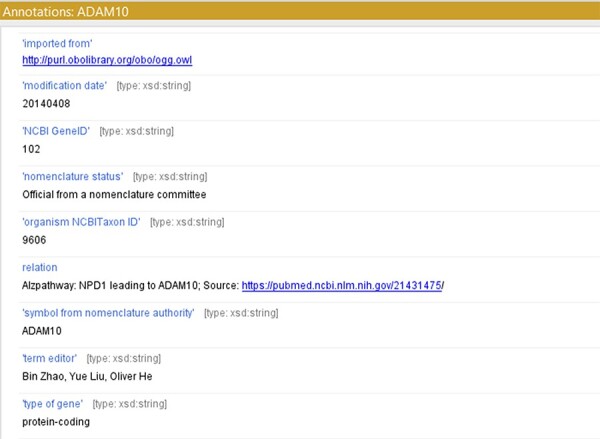
Using ‘relation’ to annotate the term with a reference source from ADMO or Alzpathway.

For creating a model with controlled vocabularies, the names of the terminologies derived from ADMO were separated into several entities. We used object properties found in Relation Ontology (RO) to express the relations described in the original ADMO entities. The ADMO model contains very general relational aspects of genes and proteins (demonstrated in Figure 2). It could be vague for the ontology users to spot molecules’ specific relationships if they are imported to the integrated ADO just as how it is presented in the ADMO ontology. To maintain clarity in the integrated model, we further investigated the specific relations of the subject and object within the terminology (within the range of risk factor genes for AD) found in ADMO through PubMed publications. Next, the most suitable object property was chosen to present the relationship between the molecules. Lastly, we annotated these terms with their original form of relation found in ADMO and the respective publication using the annotation property relation (http://purl.org/dc/elements/1.1/relation) (an example is depicted in Figure 3). The reformatted relational axioms are demonstrated in Table 2.

**Table 2.** Object properties and their relations used in ADO

Integrated ADO terminology	ADMO terminology
APOE ‘molecularlyInteractsWith’ cholesterol	APOE–cholesterol transport
APOE ‘molecularlyInteractsWith’ Amyloid beta	APOE–AmyloidB association
VLDLR ‘molecularlyInteractsWith’ APOE	VLDLR–APOE association
Amyloid precursor protein (APP) ‘molecularlyInteractsWith’ SORL1	APP–SORL1 association
CLU ‘molecularlyInteractsWith’ amyloid beta	Amyloid B–CLU dissociation; amyloid B–CLU association_Brain
LRP8 ‘molecularlyInteractsWith’ APOE	LRP8–APOE association
Protectin D1 ‘positivelyRegulates’ ADAM10	Protectin D1 leading to ADAM10

The object properties were derived from RO. Implementation of these properties enables transfer of the knowledge from ADMO to ADO while maintaining controlled vocabulary.

Abbreviations: A disintegrin and metalloproteinase domain-containing protein 10, ADAM; CLU, clusterin; LRP8, low-density lipoprotein (LDL) receptor–related protein 8; SORL1, sortilin-related receptor 1; VLDLR, very low-density lipoprotein receptor.

### Text mining bins

**Figure 4.**
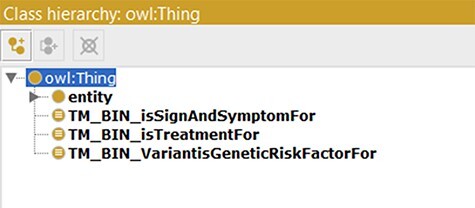
Classes used as text mining bins.

As a model tailored for text mining purposes, several text mining bins were created in the updated ADO ontology. This was achieved through creating object properties connecting to the subject of the model (AD, http://purl.obolibrary.org/obo/DOID_10652) (shown in Table 3). Here, we illustrate three object properties to connect the knowledge about AD, including AD treatment (isTreatmentFor), genetic risk factors (VarantisGeneticRiskFactorFor) and signs and symptoms (isSignAndSymptomFor). Subsequently, three classes were generated and set ‘as equivalents to’ the ‘usage of VarantisGeneticRiskFactorFor’, ‘isTreatmentFor’ and ‘isSignAndSymptomFor’. In our use cases, these classes function as bins and can be used as taggers/filters during text mining (shown in Figure 4).

**Table 3.** Original object properties used for linking concepts

Object property	Domain	Range
isAnatomicalEntityFor	Anatomical entity	AD
isCellularProcessFor	Cellular process	
isClassificationFor	AD	
isDiagnosisFor	Blood measurement, cerebrospinal fluid measurement, computed tomography, fluorine-18-fluorodeoxyglucose (FDG)-positron emission tomography, functional magnetic resonance imaging, measurement method, Mini-Mental status examination, National Institute of Neurological and Communicative Disorders and Stroke (NINCDS) and the Alzheimer’s Disease and Related Disorders Association (ADRDA) criteria for AD, past medical history, physical examination	
isModelFor	Biological model	
VarantisGeneticRiskFactorFor	ABCA7, ADAM10, ADAMTS4, ALPK2, APH1B, APOE, BIN1, CASS4, CD2AP, CELF1, CLNK, CLU, CNTNAP2, CR1, EPHA1, FERMT2, HESX1, HLA-DRB5, INPP5D, KAT8, MEF2C, MS4, NME8, PICALM, PTK2B, SLC24A4, SORL1, ZCWPW1	
isRiskFactorFor	Head injury, age, cholesterol, diabetes mellitus(disease), family history, heart disease, homocysteine, hypertension, obesity, stroke	
isSignAndSymptomFor	Symptom	
isTreatmentFor	Central nervous system drug, treatment	

Object properties were implemented in ADO for demonstrating the interconnectivity between key concepts and AD.

Abbreviations: A disintegrin and metalloproteinase domain-containing protein 10, ADAM; APP, amyloid precursor protein; CLU, clusterin; SORL1, sortilin-related receptor 1.

### Application

**Figure 5.**
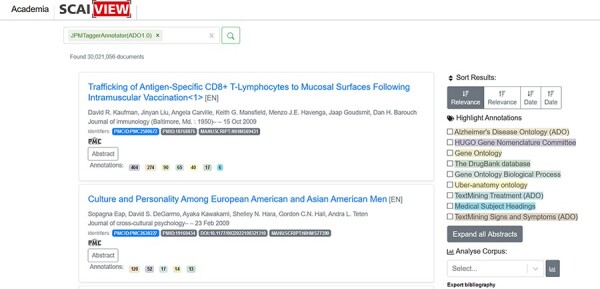
Number of documents extracted through applying the ‘ADO’ tagger.

**Figure 6.**
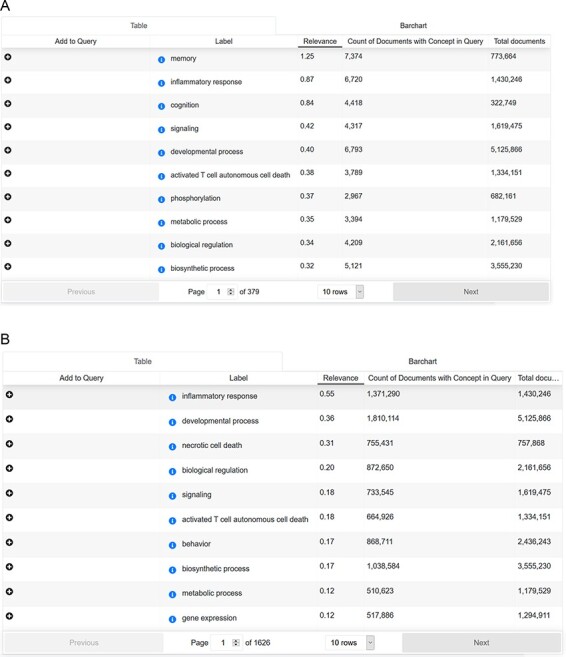
(A) Cross-analysis of AD-related symptoms and cellular processes. (B) Cross-analysis of AD-related symptoms and cellular processes.

ADO ontology could be used in many application scenarios. This paper demonstrates how ADO assists researchers in extracting textual information via text mining. As described in the Methods section, text mining models were first generated using ADO ontology. These models were integrated into the semantic search engine Academia SCAIView (https://academia.scaiview.com) and could be selected as taggers to annotate texts. In total, the search engine accesses more than 36 million documents from the MEDLINE database, of which more than 18 million are annotated. Using the ADO ontology as a search filter results in a total of 30 021 056 documents retrieved [many documents were retrieved using ADO ontology as a tagger due to the wide range of concepts (and their synonyms) included in the upper and intermediate levels of ADO ontology]. Other sub-ontologies derived from ADO, such as ‘signs and symptoms’, ‘genetic risk factors’ and ‘treatment’, can also be used as taggers. We give several examples of how the taggers can be utilized as follows:

- Publication filtering based on individual taggers
  - The integrated ADO-based models can be incorporated as bins tagging the abstracts of the documents that match the context. When we applied the whole ADO as the text tagger, a number of documents were retrieved, shown in Figure 5. While applying the other taggers, different numbers of documents were obtained (86 877 documents for the genetic risk factor tagger, 4 985 933 documents for the signs and symptoms tagger and 8 907 809 documents for the treatment tagger).
- Cross-analysis between AD-related symptoms and cellular processes
  - To answer this question, we searched with the concept ‘Alzheimer Disease’ (Medical Subject Headings (MESH) term) in combination with the ‘signs & symptoms’ filter. ‘Signs and symptoms’ were analyzed for their frequency of occurrence and overlapping mentions. Subsequently, ‘signs and symptoms’ results were applied as a search paradigm (in combination with the concept ‘Alzheimer Disease’ and without) and analyzed for ‘biological process’ mentions (*Source:* Gene Ontology). For each sign and symptom pattern, we could identify the cellular processes ranked according to their frequency of occurrence. Interestingly, there is an overlap between AD-related mentions and non-AD-focused signs and symptoms patterns (shown in Figure 6).
- Mentions of drugs in the context of AD-related signs and symptoms
  - We applied the ‘signs and symptoms’ filter and analyzed the resulting corpus concerning DrugBank annotations. The three most mentioned drug compounds in our search context were methamidophos, bifenthrin and flufenoxuron. The complete query result is available in Supplementary Table S1.
- Biological processes/pathways that might play a role in AD

As the ‘genetic risk factor’ tagger was applied in SCAIView, the corpus was then analyzed for relevance in ‘gene ontology biological processes’ annotations. The analyzed corpus was filtered using ‘pathway’ and its synonyms. We added each pathway term to the query and noted the number of literature studies extracted. As exhibited in Supplementary Table S2, the Wnt signaling pathway and Notch signaling pathway are the most intensively researched pathways concerning the genetic risk factors found in AD patients.

## Discussion

The ADO ontology integrates AD-related knowledge acquired from multiple sources. Many knowledge terminologies of the integrated ADO were collected from the previous version of ADO and the ADMO ontology. The core concepts related to Alzheimer’s research were retained, and redundancy was avoided. Integrating new knowledge on top of them has made the updated version more valuable for knowledge retrieval. The successful construction and its approval from the OBO Foundry enlighten several future perspectives. This section discusses the usage of the ADO ontology, future perspectives with ADO, challenges and its limitations.

### Using ADO ontology and its contribution to the community

The integrated ADO ontology is the first attempt at interoperable ontology in the AD domain. The integrated ADO enables the integration of heterogeneous data sources by providing a common vocabulary and framework, thus harmonizing the data from different origins. As an OBO ontology, this ontology is now available on the OLS platform after acceptance from the foundry and the integrated ADO will be maintained in the upcoming 3 years since its official release. Our work not only contributes to the domain ontology community with AD-oriented terminologies but also represents the current understanding of this disease as a condensed and controlled knowledge model, allowing for various research purposes.

### Application of ADO

As illustrated in the previous sections, ADO could be used as a text filter, helping obtain relevant data when different taggers are applied. Corpora analysis with distinct taggers enables an understanding of more complex questions. Just as the use cases given earlier, these annotators could be employed for analyzing the relevance of terminologies, research intensity, connection between different knowledge aspects or filtering out desired contents in the Alzheimer’s context. There are more possibilities beyond the examples given in this work when using ADO as text mining taggers, such as identifying relational aspects in the ontology or fuzzy search. We welcome and encourage researchers to use ADO for more possibilities.

### Future perspectives with ADO

Although the text mining models generated using ADO for literature filtering were the only application discussed in this paper, more possibilities with ADO ontology could be explored. For example, ADO-based clinical supporting systems could significantly improve data exchangeability in clinical usage. The free texts the clinicians give take much work to standardize. As one of the options, integrated ADO ontology could be used for tagging specific words from the texts. The tagged words and phrases are restructured with controlled vocabularies and harmonized textual data across multiple clinical facilities. This application could enhance data shareability and exchangeability within the community. Other possibilities with the integrated ADO ontology include knowledge alignment, ontology embeddings or model reasoning. Alignment between the integrated ADO and other semantic models in this disease domain could help researchers explore comorbidities; ontology embeddings with ADO, on the other hand, shed light on potentially solving learning problems in Artificial Intelligence (AI) models; model reasoning with ADO enables logical deductions and drawing conclusions based on the defined relationships and rules within the ontology.

### Challenges

Building an integrated knowledge model could be challenging in several ways, and we encountered several obstacles along the process. The first challenge we faced was the scope and focus of the ontology. During the selection of terms, we realized that the range of the original ADO and ADMO was broad, containing terms from demographics, specific laboratory models and single-nucleotide polymorphisms. Deciding the terminologies needed for the integrated version was difficult. After careful inspection of the two source ontologies, our team decided to maintain the integrated ontology small and informative, leaving out the terminologies with high specificity and hierarchically functional entities (those without obvious contributions to the knowledge aspects and mainly used for classifying the downstream entities).

Additionally, we struggled to find the most appropriate relations for the ADMO terminologies. The relations described in ADMO were generalized without mentioning the specific type of molecular interaction. The initial attempt was to find the specific interaction type in the RO and connect the molecular entities accordingly. However, it took work to distinguish the most appropriate relations, given that RO is a relatively small ontology and only provides simple object properties to illustrate their relationships. This eventually became a trade-off problem between interoperability and defining detailed relations between entities by ourselves. As a result, we chose to stick with the RO for the relational presentation and maximize the interoperability.

Moreover, reusing parts of ontology is often necessary, but enacting reuse is a challenge (e.g. finding the right concepts, sub-trees from the right ontologies or finding the appropriate extraction routines). We dedicated efforts to finding the equivalent terminologies on OLS for the entities derived from the original ADO and the ADMO ontology to maintain the level of orthogonality (33) and interoperability. Specifically, we attempted to closely investigate the definition of the terminologies that might have the same meaning yet are named differently. Reusing existing entities with the same naming was also carefully carried out, given that the definition and meaning of the entities could differ when found in distinct ontologies and contexts.

### Limitations

Several points with ADO need to be addressed when more advanced methodologies are developed. For example, maintenance of ADO could be time-consuming since manual curations and knowledge selection are required. Moreover, ADO has imported terminologies from multiple existing ontologies, and these terms contribute to a big portion of the integrated ADO. It could be problematic if the original ontology maintainers update the entities imported into the integrated ADO ontology in the future. In that case, ADO will not be updated correspondingly (with the current version of Protégé). This problem is a common issue in the ontology community and awaits improvement.

Apart from the maintenance challenge, there is also a limitation in what the ADO covers. We intentionally omitted information about laboratory models like mouse strains to keep the core focus of this ontology. However, this means that the integrated ADO may not be suitable for applications that require detailed information about specific genetic variants or intricate laboratory experiments. It is also essential to recognize that creating the integrated ADO ontology involves some subjectivity on the part of the ontology creators, similar to what happens when building other models. We sincerely welcome the ideas that could contribute to the model’s improvements from the ontology community.

The use cases of ADO illustrated in the ‘Application’ section were limited by the models’ mining power, provided that only synonyms of the target entities were included as taggers. Given the fact that people face difficulties when using other textual annotations as the tagging annotation such as definitions, more advanced techniques (i.e. tokenization of definition texts combined with machine learning) are required in order to process the free-text data and thus expand the annotations of each concept and increase the mining power.

Finally, we have noticed that the ontologies aiding in AD research have been subject to problems such as low utilization, and many of these ontologies are no longer updated (34). The primary reason causing this issue was conflicts in the names of the terminologies and inconsistent references, among others. Alba Gomez-Valades *et al.* (34) suggested that reusing the classes from existing ontologies could help address this issue. Despite significant efforts being put into reusing entities from the other existing OBO ontologies, the integrated ADO contains many classes with long phrases (such as ‘preservation of independence in functional abilities’). These symptomatic phrases play an important role in the clinical assessment of AD patients; however, they could be difficult for data harmonization as other words might be used to express the meaning in other contexts. The poor generalization and orthogonality of these terms might hinder the use of ADO in minor cases. We encourage researchers and clinicians to use shared and controlled vocabularies throughout AD research and diagnostics, promoting the AD research further.

## Materials and Methods

In this section, the specific workflow and methods used in building ADO ontology are discussed.

### Construction of ADO

**Figure 7.**
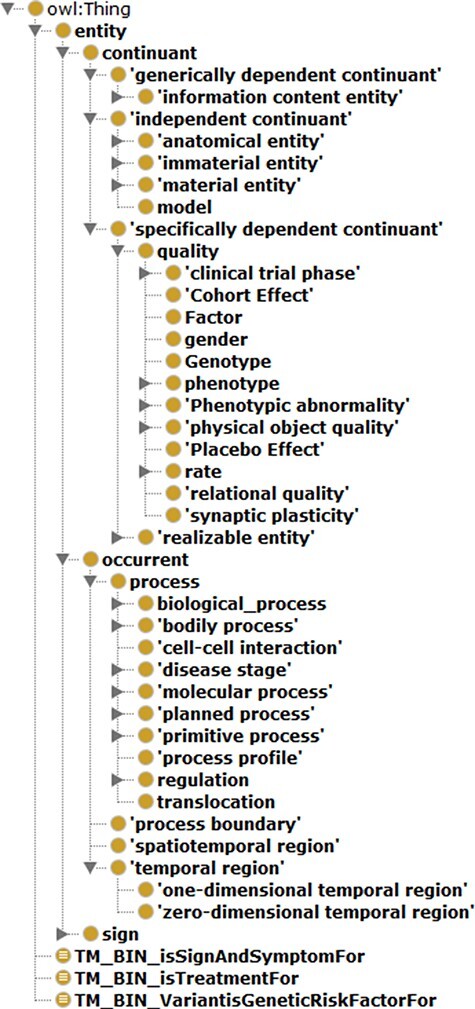
General hierarchical view of ADO ontology.

Protégé (Version 5.0) (35) was the tool used to construct ADO. This work’s scale and range were first defined, and the corresponding terms from the BFO upper-level ontology were imported for architecturing ADO. In particular, only selective terms from BFO were imported, providing the necessary scaffolding and avoiding overextension. The hierarchy was expanded after that with intermediate classes from the disease domains such as neurological disease and neurological and physiological symptoms. The general hierarchical view of BFO-supported ADO is depicted in Figure 7.

**Figure 8.**
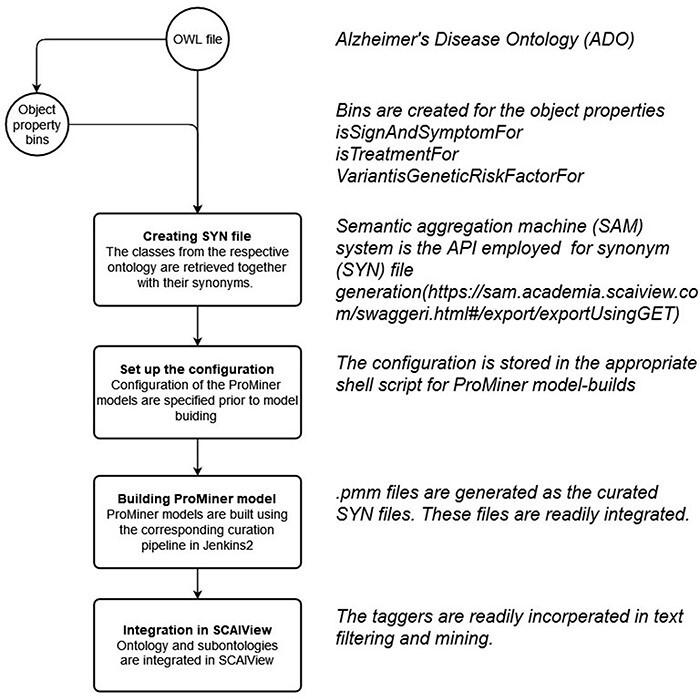
Schematic view of integration of ADO in SCAIView.

### Knowledge Collection and Import

Provided by the European Molecular Biology Laboratory European Bioinformatics Institute, OLS serves as a portal to the ontology databases where the biological terminologies and their annotations could be retrieved. The former ADO contains knowledge related to AD. However, the entities lack the expression with controlled vocabulary. For the sake of achieving an interoperable ontology and improving ontology networking in a unified standard, we first investigated each term derived from the former ADO ontology. These terms derived from the previous version of ADO were criticized for its relevance, interoperability, usage in text mining, availability in OBO ontologies and if adding the term leads to redundancy and obsolescence. In addition, multiple original classes were created along with those imported and annotated with a label, definitions, synonyms and sources. The popular and enriched AD research databases were particularly selected as knowledge inputs while defining new terms.

### Ontology usage optimization

In many cases, one single term can be found in many ontologies and its annotations vary according to the ontology where it is stored. In this work, a specialized ontology in the AD domain is intended. Hence, the entities were criticized for their abundance of annotations and relevance to our study domain. As one of the selection criteria, we selected the terms with the most accurate and abundant annotations out of the ontologies closely linked to biomedicine, neurodegenerative disease or related fields. More specialized ontologies were preferred over the ontologies such as Ontology for miRNA Target, National Cancer Institute. Thesaurus OBO Edition and Ontology for Biomedical Investigations, often with a deficit in the specificity of their class definitions. In addition, specific proteins are found overexpressed/underexpressed in AD *in vivo* models and patients. It remains unclear whether they, or at least a great portion, participate in the disease’s pathogenesis and progression. These phenotypic studies suggest abnormally regulated genes in the underlying disease although the mechanism might remain unknown. We investigated the genes encoding these proteins to achieve a higher coverage during the text mining process. We included the encoding genes instead of the proteins because the gene names in publications often denote proteins. This protein-gene conversion was accomplished by mapping our data to GeneCards.

### Additional knowledge from external sources

A number of web sources were used for enriching ADO with the up-to-date knowledge including Alzforum (https://www.alzforum.org), Alzheimer’s association (https://www.alz.org) and PubMed (36–39). We browsed the two websites and explored the relevant knowledge in AD research. The stages of AD progression, risk factor genes, possible treatments and necessary diagnostic tools were all considered essential knowledge, and we attempted to include all these aspects in ADO ontology. Knowledge integration follows as the next step. To integrate these data, we selected the most common name as the label and looked for definitions provided by publications. Finally, we annotated these terms with the source link from which they are defined.

### Alzpathway

AD-related information is available in Alzpathway, detailing the interaction of biomolecules and their upstream/downstream relations via a DM. In order to increase interoperability of the terms in our ontology, we deliberately filtered out the relations involving the imported risk factor genes and reformulated them. ‘Apolipoprotein E (APOE)–AmyloidB association’ exemplifies the format of ADMO entities. The selected entity has multiple constituents, including a gene, another material entity (lipid, protein or gene), and their relation. To improve interoperability and quality of text retrieval from text mining, the most appropriate relations available from RO, an OBO ontology, were selected to connect the subject and object. The original terms were restructured in a ‘Domain ObjectProperty Range’ format.

### Synonym file creation and data integration in SCAIView

Within the integrated ADO ontology, object properties such as ‘is treatment for’, ‘Variant is genetic risk factor for’ and ‘sign and symptoms for’ are used for connecting respective concepts and the term AD. Subsequently, classes equivalent to the respective object properties were created (‘TM_BIN_isSignAndSymptomFor’, ‘TM_BIN_isTreatmentFor’ and ‘TM_BIN_VariantisGeneticRiskFactorFor’). These classes and the complete updated ADO ontology were all used as taggers through which documents and texts were put in corpora. As depicted in the flowchart in Figure 8, the synonym files were generated by extracting the synonyms from corresponding classes and arranging them in a format read by ProMiner (40). Synonyms of classes can be spotted by the semantic aggregation machine from SCAIView, extracting the related synonyms through various synonym-related annotation properties (e.g. hasExactSynonym and hasSynonym). ProMiner is a software package designed for terminology recognition in the life science field, and its models were subsequently generated from the synonym files, finally enabling integration of the terms in SCAIView for applications.

## Supplementary Material

baad085_Supp

## Data Availability

The update version (2.0.1) of ADO ontology can be accessed via the github repository: https://github.com/Fraunhofer-SCAI-Applied-Semantics/ADO.
